# Acute liver injury and diffuse subcutaneous pneumatosis following compound paracetamol and amantadine hydrochloride tablets poisoning: a case report

**DOI:** 10.3389/fphar.2025.1503449

**Published:** 2025-04-25

**Authors:** Zhiwen Zhao, Bo Wang, Lanlan Cai, Xuefang Liu, Zhicheng Fang

**Affiliations:** ^1^ Department of Emergency Medicine, Taihe Hospital, Hubei University of Medicine, Shiyan, Hubei, China; ^2^ Department of Pediatric Surgery, Taihe Hospital, Hubei University of Medicine, Shiyan, Hubei, China

**Keywords:** paracetamol, amantadine, acute liver injury, diffuse subcutaneous pneumatosis, prognosis

## Abstract

**Introduction:**

Severe acute poisoning from Compound paracetamol and Amantadine hydrochloride tablets can result in various complications, including acute liver injury, central nervous system reactions, and gastrointestinal symptoms.

**Case summary:**

A 15-year-old previously healthy female ingested 108 Compound paracetamol and Amantadine hydrochloride tablets. She presented to the emergency department with severe nausea, vomiting, abdominal pain, and distension, along with confusion. Emergency interventions, including gastric lavage and endotracheal intubation for respiratory support, were initiated. She was subsequently transferred to the intensive care unit for advanced life support, including nasogastric tube, hemoperfusion, and hemodialysis. During treatment, she developed coagulation abnormalities, liver injury, acute gastric mucosal bleeding, electrolyte imbalances, and psychiatric symptoms. A computed tomography (CT) scan revealed diffuse subcutaneous pneumatosis extending to the neck, anterior chest wall, thoracic cavity, and mediastinum. Following aggressive treatment, the patient was successfully discharged on the seventh day after the overdose.

**Conclusion:**

Acute liver injury and diffuse subcutaneous pneumatosis affecting multiple regions, caused by the ingestion of a large quantity of Compound paracetamol and Amantadine hydrochloride tablets, is a rare occurrence. With early gastric lavage, blood purification, and supportive therapy, the patient was successfully treated and discharged.

## Introduction

Compound paracetamol and Amantadine hydrochloride tablets are a commonly used combination preparation for the treatment of influenza. Each tablet contains 250 mg of acetaminophen (APAP), 100 mg of amantadine hydrochloride, 10 mg of artificial bovine flavor, 15 mg of caffeine, and 2 mg of chlorpheniramine maleate. Excessive intake of APAP can lead to serious complications, including acute liver injury, liver failure, and even death (Yoon et al., 2016), primarily characterized by liver failure caused by centrilobular necrosis. According to statistics, approximately 300 people in the United States died from APAP poisoning in 2020 (Gummin et al., 2020). Ingesting doses greater than 12 g or having serum APAP concentrations above the therapeutic threshold on the APAP nomogram are associated with an increased risk of hepatotoxicity (Wong and Graudins, 2017). Currently, N-acetylcysteine (NAC) is the most commonly used antidote for APAP poisoning, typically administered intravenously (Saccomano, 2019). Although most cases of APAP poisoning can be treated with NAC, patients who have ingested large quantities may still require hemodialysis (Gosselin et al., 2014).

Massive gas accumulation in tissues by Compound paracetamol and Amantadine hydrochloride tablets, especially in cases of drug toxicity, is an exceptionally rare complication, and its precise mechanism remains unclear. In this paper, we present the case of a 15-year-old previously healthy female who developed acute liver injury, coagulation abnormalities, electrolyte imbalances, and psychiatric symptoms following the ingestion of 108 Compound paracetamol and Amantadine hydrochloride tablets. A computed tomography (CT) scan revealed diffuse subcutaneous pneumatosis extending to the cervical region, anterior chest wall, thoracic cavity, and mediastinum. After aggressive treatment, the patient was successfully discharged from the hospital.

This case report aims to highlight the rare coexistence of acute liver injury and diffuse subcutaneous pneumatosis following Compound Paracetamol and Amantadine Hydrochloride tablet poisoning. Further research is needed to elucidate the underlying mechanisms contributing to massive gas accumulation in drug poisoning, thereby providing clinicians with valuable guidance to identify, prevent, and intervene in such rare complications.

## Case summary

The patient, a healthy 15-year-old female, presented to the emergency department with severe nausea, vomiting, abdominal pain, abdominal distension, and confusion, which began 1 hour after ingesting 108 tablets of Compound Paracetamol and Amantadine Hydrochloride.

**FIGURE 1:**
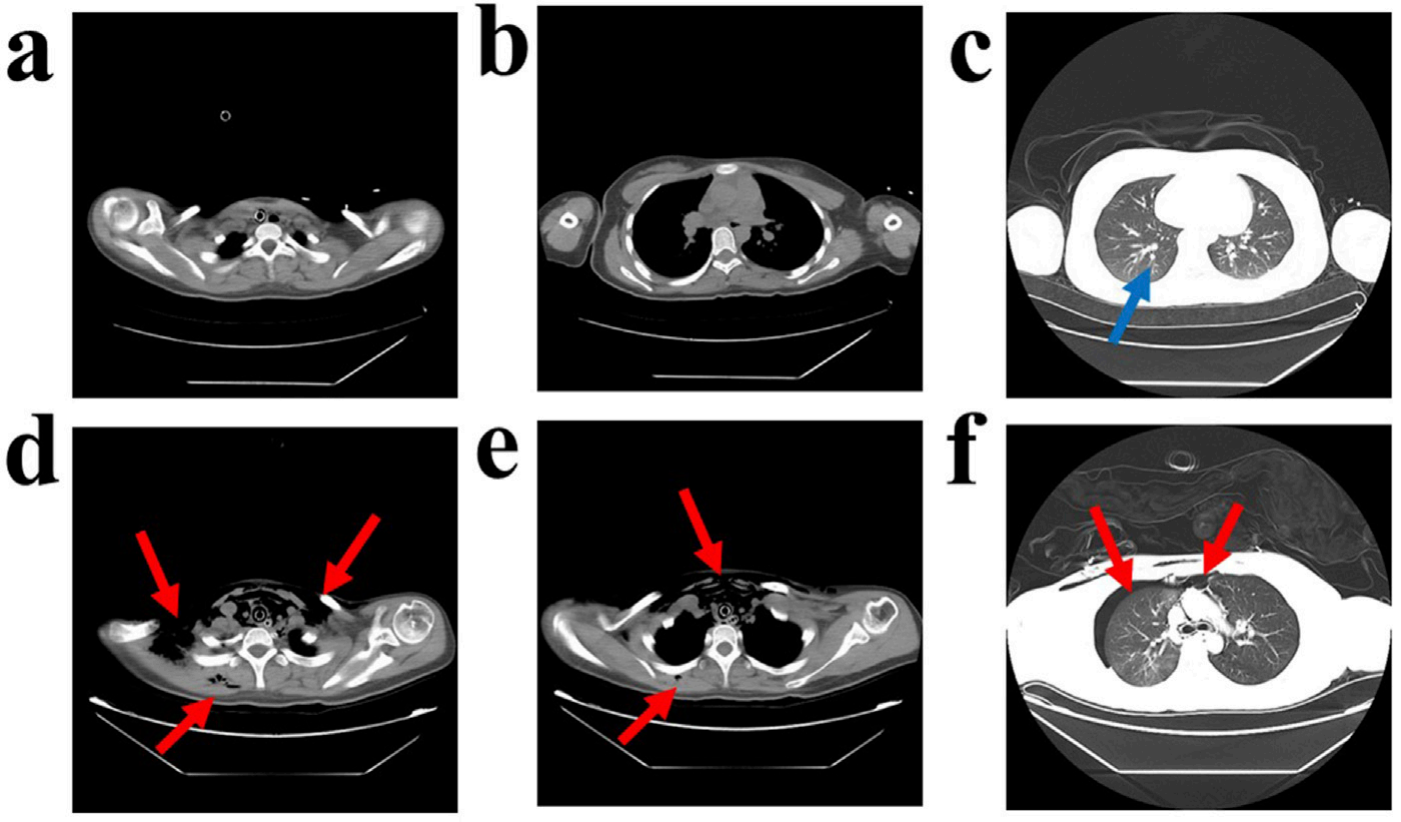
Computed Tomography Scan of the Patient’s Chest. A computed tomography (CT) scan of the chest taken on the first day of admission revealed minor inflammation in the lower lobe of the left lung, as indicated by the blue arrows **(a–c)**. The same CT scan also showed diffuse air accumulation in the bilateral neck, shoulders, anterior chest wall, thoracic cavity, mediastinum, and subcutaneous air in the right dorsum, with approximately 20% compression of the right lung. Additionally, multiple patchy ground-glass opacity areas were observed in the right upper lobe and the lower lobes of both lungs, with indistinct borders, as indicated by the red arrows **(d–f)**.

On physical examination, her vital signs were recorded as follows: body temperature 36.2°C, heart rate 88 beats per minute, respiratory rate 22 breaths per minute, blood pressure 109/66 mmHg, and oxygen saturation 86%. As shown in Table 1, arterial blood gas analysis revealed respiratory insufficiency, acidosis, hypernatremia and hypokalemia. She was immediately intubated for respiratory support and underwent gastric lavage, during which no drug tablets were found. An electrocardiogram (ECG) revealed sinus rhythm with frequent and paired ventricular premature beats, along with T-wave changes (low and flat in leads II, III, aVF, V5, and V6). A chest CT scan showed minor inflammation in the lower lobe of the left lung (see Figures 1a–c).

**TABLE 1:** Arterial blood gas analysis showed the following results.

Parameter	Value	Reference value range
PH	7.22	7.35–7.45
PaCO_2_ (mmHg)	22.2	35–45
PaO_2_ (mmHg)	58	80–100
Na^+^ (mmol/L)	162.8	135–145
K^+^ (mmol/L)	2.4	3.5–5.5

The patient was subsequently transferred to the emergency ICU for advanced life support. Invasive ventilator-assisted ventilation was initiated with the following parameters: P-A/C mode, PS 20 cmH_2_O, PEEP six cmH_2_O, respiratory rate 16 breaths/min, and FIO_2_ 60%. A nasogastric tube was placed for gastrointestinal decompression, correct internal environmental disturbances, and intravenous NAC was administered. The initial dose consisted of 250 mL of 5% dextrose injection with NAC 7,000 mg over 60 min, followed by 250 mL of 5% dextrose injection with NAC 3,500 mg every 4 h, for a total duration of 72 h. Oral glutathione tablets (400 mg per dose, three times daily) were also administered. The patient underwent one haemoperfusion session and two haemodialysis filtration treatments. We recommended drug metabolism and toxicology tests to the patient’s family, but they declined.

As shown in Table 2, biochemical examination on the second day of admission (36 h post-poisoning) indicated hepatocellular damage and mild coagulation abnormalities. Palpation revealed swelling in the bilateral neck and right anterior chest area, which produced subcutaneous gas movement when pressed. A sound similar to the twisting of hair could be heard on auscultation. A chest CT confirmed diffuse subcutaneous pneumatosis in the bilateral neck, shoulders, anterior chest wall, thoracic cavity, mediastinum, and right dorsum, along with approximately 20% compression of the right lung. Additionally, multiple areas of ground-glass opacity with blurred borders were observed in the upper lobe of the right lung and the lower lobes of both lungs (see Figures 1d–f). To treat the diffuse subcutaneous pneumatosis, a sterile syringe was used to puncture the skin and release the gas.

**TABLE 2:** Trends in hepatic and renal function and coagulation indices in patients undergoing treatment.

Coagulation function	1 h post- poisoning	12 h post- poisoning (Day 1)	36 h post- poisoning (Day 2)	60 h post- poisoning (Day 3)	Reference value range
ALT (U/L)	11.2	106.7	64.2	20.2	0–50
AST (U/L)	20.5	92	31.5	14.9	0–40
γ-GT (U/L)	12.2	19.1	12.1	18.4	0–60
ALP (g/L)	105.6	109.2	85.2	75	40–150
TP (umol/L)	80.27	77.14	55.64	56.34	40–55
TBil (umol/L)	8.79	19.78	9.65	6.42	2.0–20.4
CBil (umol/L)	1.23	6.8			0–6.8
Urea (mmol/L)	4.96	2.75	1.07	2.66	1.7–8.3
Cr (umol/L)	42.49	26	43.6	36.3	44–120
UA (umol/L)	252.6				208–428
PTA	87	55	59		
PT(s)	11.9	17	16		11–14
INR	1.11	1.6	1.5		0.8–1.2
APTT(s)	28.8	34.8	36.5		20–40
Fig (g/L)	4.13	3.58	4.52		2–4
TT(s)	14.3	14.4	14		16–18
UA (umol/L)	252.6				208–428

ALT, alanine aminotransferase; AST, aspartate aminotransferase; γ-GT, γ-Gamma Glutamyl Transpeptidase; ALP, alkaline phosphatase; TP, total protein; Tbil, Total Bilirubin; Cbil, Conjugated Bilirubin; Cr, Creatinine; UA, uric acid; PTA, prothrombin activity; PT, prothrombin time; INR, international normalized ratio; APTT, Activated Partial Thrombo-plastin Time; Fig, Fibrinogen; TT, thrombin time.

On the third day of hospitalization (60 h post-poisoning), the patient was successfully weaned from mechanical ventilation, and the hepatocellular damage had improved. During the hospitalization period, the patient experienced intermittent irritability and emotional breakdowns.

On the sixth day, an intracranial magnetic resonance imaging (MRI) scan and an electroencephalogram (EEG) were performed, test results showed normal. There was a significant reduction in subcutaneous pneumothorax, and both coagulation and liver functions had returned to normal. The patient was discharged on the seventh day of hospitalization.

## Discussion

This case highlights the rare but severe complication of diffuse subcutaneous pneumatosis following poisoning by a combination of paracetamol and amantadine hydrochloride tablets, emphasizing the importance of early detection and intervention. Paracetamol and amantadine hydrochloride tablets are widely used antiviral drugs in China for treating influenza. Paracetamol is a commonly used antipyretic and analgesic, particularly during the influenza season (Lee et al., 2024). Amantadine, first used in the 1950s for its antiviral properties, was later found in the late 1960s to be therapeutic for movement disorders related to Parkinson’s disease (Zhang et al., 2022). Artificial oxalis, caffeine, and chlorpheniramine maleate are included in the formulation for their unique pharmacological effects.

Diffuse subcutaneous pneumatosis due to drug overdose is an extremely rare complication to poisonings. Trichostatin poisoning can lead to multi-organ failure and gas accumulation in the portal venous system and mesenteric vessels, which typically signals a poor prognosis (Yu et al., 2024). Immune checkpoint inhibitors (ICIs) can also cause intestinal emphysema in rare cases, with drugs like pembrolizumab known to trigger gas accumulation in the abdomen, retroperitoneum, mediastinum, and pneumothorax (Calenda et al., 2024). Prolonged use of opioids (e.g., heroin) inhibits gastrointestinal motility, leading to massive pneumoperitoneum and perforation (Ben et al., 2024). Similarly, olanzapine has been associated with these effects (Rodrigues et al., 2018). In patients with alcoholism, an overdose of alcohol mixed with drugs can cause not only acute gastric mucosal necrosis but also portal pneumoperitoneum (Silvers et al., 2022). Cocaine abusers may develop diffuse subcutaneous emphysema, pneumothorax, and pulmonary hemorrhage (Atmaca Temrel et al., 2015).

As previously reported, compound Paracetamol and Amantadine hydrochloride tablets may cause additional lung injuries beyond diffuse subcutaneous pneumatosis in the thorax and mediastinum. In patients with end-stage renal disease, a sudden onset of respiratory failure and Acute Respiratory Distress Syndrome (ARDS) has been observed after administering 300 mg of amantadine per day without an apparent cause (Cattoni and Parekh, 2014). The likely explanation for this is the significant accumulation of the drug in the body due to impaired renal function. Our patient developed diffuse subcutaneous pneumatosis in the anterior chest wall, thoracic cavity, and mediastinum after ingesting a large quantity of Compound Paracetamol and Amantadine hydrochloride tablets. The diffuse subcutaneous pneumatosis resolved with conservative treatment; however, its exact cause remains uncertain. Building on previous studies regarding gas accumulation caused by drug intoxication (Calenda et al., 2024; Ben et al., 2024; Rodrigues et al., 2018), we hypothesize that diffuse subcutaneous pneumatosis resulting from compound paracetamol and amantadine hydrochloride tablet poisoning may be linked to the following factors. First, the administration of large doses of these drugs may reduce gastrointestinal peristalsis, leading to gas accumulation in the intestinal lumen, which then passes through the intestinal mucosal barrier into the bloodstream or subcutaneous tissues. Second, recent studies have indicated that APAP exposure could have potentially toxic effects on the lungs (Grayck et al., 2025), which may alter the tone and permeability of the pulmonary blood vessels. This, in turn, increases the permeability of the alveolar walls, allowing gas to permeate into the interstitial and subcutaneous tissues. More importantly, toxicity from high drug doses may weaken the alveolar walls, increasing the risk of alveolar rupture. When the alveoli rupture, air leaks into the surrounding tissues, culminating in diffuse subcutaneous pneumatosis.

In our patient’s case, routine gastric lavage was performed after the ingestion of a large quantity of Compound paracetamol and Amantadine hydrochloride tablets, but no tablet residue was observed, suggesting that the drug had already been absorbed. We administered NAC in combination with glutathione to protect liver function, and we facilitated drug metabolism and clearance through hemoperfusion and hemodialysis. These therapeutic interventions were crucial for the patient’s complete recovery. Therefore, further in-depth studies are needed to elucidate the pharmacological activities of amantadine.

## Conclusion

This case report highlights a rare instance of acute hepatic injury and diffuse subcutaneous pneumatosis affecting multiple regions, caused by the massive intake of Compound paracetamol and Amantadine hydrochloride tablets. It suggests that this condition may be related to the combined effects of the drug’s multiple components or individual patient differences. With early intervention through gastric lavage, blood purification, and supportive therapy, the patient was successfully treated and ultimately recovered, being discharged from the hospital. Future studies should further investigate the mechanisms underlying multi-system responses following Paracetamol and Amantadine poisoning, with the aim of optimizing therapeutic regimens and reducing the morbidity, mortality, and complication rates associated with such poisoning events.

## Data Availability

The original contributions presented in the study are included in the article/supplementary material, further inquiries can be directed to the corresponding author.
